# A case report: Intermittent catheterization combined with rehabilitation in the treatment of carbapenem-resistant *Klebsiella pneumoniae* catheter-associated urinary tract infection

**DOI:** 10.3389/fcimb.2022.1027576

**Published:** 2022-11-09

**Authors:** Lihua Shen, Heng Wu, Huiying Chen, Yan Jiang

**Affiliations:** ^1^ Department of Infectious Diseases, Sir Run Run Shaw Hospital, Zhejiang University School of Medicine, Hangzhou, China; ^2^ Key Laboratory of Microbial Technology and Bioinformatics of Zhejiang Province, Hangzhou, China; ^3^ Regional Medical Center for National Institute of Respiratory Diseases, Sir Run Run Shaw Hospital, Zhejiang University School of Medicine, Hangzhou, China

**Keywords:** intermittent catheterization, carbapenem-resistant *Klebsiella pneumoniae*, catheter-associated, urinary tract infection, rehabilitation

## Abstract

Catheter-related urinary tract infections, especially those caused by multidrug-resistant (MDR) bacteria, are extremely difficult to treat due to limited therapeutic choices. Therefore, removing catheters as soon as possible is pivotal to successful treatment. Herein, we report a case of catheter-related urinary tract infection caused by carbapenem-resistant *Klebsiella pneumoniae* (CRKP). Intermittent catheterization was used to reduce biofilm occurrence and exercise bladder function on the basis of an active and adequate anti-infection strategy. Simultaneously, combined with acupuncture treatment and strengthening the patient’s pelvic floor muscle training to improve urinary retention, the catheter was eventually removed to obtain autonomous urination in this patient, and this led to the successful treatment for a CRKP catheter-related urinary tract infection.

## Introduction

According to the World Health Organization (WHO), carbapenem-resistant strains in *Enterobacteriaceae* (CRE), *Pseudomonas aeruginosa* and *Acinetobacter baumannii* is considered critical, being classified as Priority 1 group because of its resistance to this last resource antibiotic class, among which *Klebsiella pneumoniae* and *Escherichia coli* are the most common (Podschun and Ullmann, 1998; WHO, 2017), becoming important pathogenic bacteria of hospital-acquired infections. Carbapenem-resistant *Klebsiella pneumoniae* (CRKP) can cause bloodstream infections, abdominal cavity infections and urinary tract infections, and these patients are often in critical condition with limited therapeutic strategies and poor prognosis (Hu et al., 2012; van Duin et al., 2013; Zhao et al., 2015). One of the principles of anti-infection therapy is to control the source of infection. However, many patients with urinary tract infections associated with catheters often cannot have their catheters removed due to underlying diseases, which brings challenges to clinical treatment. Costerton et al. (1999) clearly proposed that bacterial biofilm formation is a common pathogenic mechanism causing persistent bacterial infections. Previous studies have pointed out that the duration of indwelling catheters is a risk factor for the formation of urinary tract bacterial biofilms, the probability of urinary tract infection in patients who retain catheters for 3 days is more than 90%, and the risk of biofilm formation on the catheter wall may increase 3.55 times with each 1-day extension of the indwelling catheter time (Costerton et al., 1999; Miranda et al., 2017). Furthermore, the formation of biofilms increases the difficulty of anti-infection treatment, especially for multidrug-resistant (MDR) bacteria. Effective measures to prevent biofilm formation include strict indwelling catheterization indications that reduce unnecessary catheter use and daily evaluation of patients for early catheter removal. Therefore, successful removal of catheters is an important part of treatment for catheter-related urinary tract infections. In this case, the treatment included both active anti-infection therapy according to *in vitro* susceptibility results and an intermittent catheterization method to shorten the catheter indwelling time, which prevented bacterial biofilm formation. Combined with acupuncture and rehabilitation training to promote autonomous urination, this treatment was eventually successfully used to treat the patient’s catheter-related CRKP urinary tract infection.

## Case description

**Figure 1:**
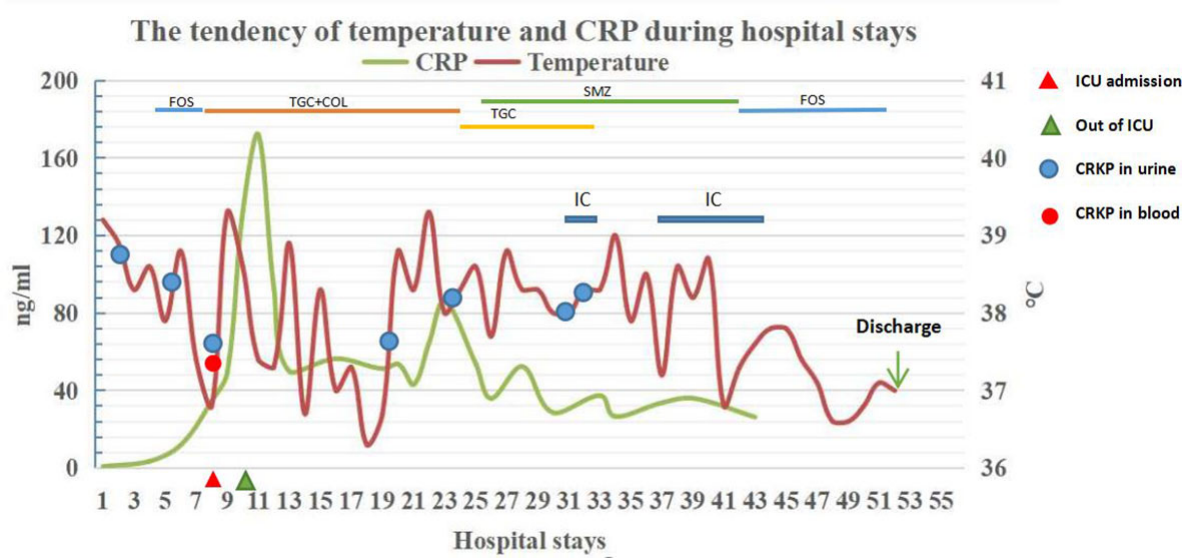
The tendency of CRP value (left Y axis) and temperature (right Y axis) during hospital stays. FOS, fosfomycin; TGC, tigecycline; COL, colistin; IC, intermittent catheterization.

A 66-year-old female with a urethral catheter was hospitalized because of persistent fever for 19 days (20 days after surgery for suppurative appendicitis). Her underlying diseases included sicca syndrome, cerebral infarction and hypertension. After admission, the patient was treated with fosfomycin ambutritol 3 g QD orally, and her body temperature and C-reactive protein (CRP) gradually decreased to normal. The catheter was removed on the 8th day after admission. Unfortunately, the patient was admitted to the ICU with advanced life support due to septic shock on the night of extubation. Both blood and urine cultures showed CRKP, which was subsequently confirmed to produce KPC carbapenemase. The patient’s anti-infection therapy included tigecycline 100 mg q12 h and polymyxin B 50wu q12 h. Her blood culture turned negative 2 days later, but the urine culture continued to grow CRKP. After the administration of polymyxin B for 16 days, an *in vitro* susceptibility test showed that the MIC of polymyxin B was 64 mg/L, which indicated high-level resistance; thus, we stopped the administration of polymyxin B use and started oral SMZco 0.96 BID. During the treatment, we tried 3 times to remove the catheter, but it had to be reset due to urinary retention. The patient was still unable to urinate autonomously and relied on the persistent use of urethral catheters, leading to the failure of controlling the infection source. The patient’s routine urinalysis was reexamined: the white blood cell (WBC) count was 22.4/UL, the bacteria count was 23564.9/UL, and nitrite was negative. The temperature of the patient did not drop into the normal range. Then, intermittent catheterization was performed based on the use of SMZco. At 10 am every day, urine was taken from the catheter for culture and routine urinalysis. Then, the catheter was removed, and the catheter tip was submitted for culture. Aseptic urethral catheterization was performed again within 2-4 hours, and catheterization was performed until 10 am on the next day. A size 12CF catheter was selected to reduce the urethral mucosal damage. The patient was given a reasonable amount of drinking time and amount of water, with a total daily intake of 2000 ml, 200 ml between 10 am and 13 pm, and the patient could have water intake at other times as required. At the same time, the acupuncturist in the department of rehabilitation administered acupuncture treatment once a day. The patient was required to perform pelvic floor muscle function training with the decubitus position, actively contracting and clamping the anus and urethral orifice, by contracting and relaxing the muscles for 5-10 seconds, which was repeated 5-10 times for 1 cycle. Two cycles were performed each day in the morning, mid-day and evening. The patient’s intermittent catheterization lasted for 9 days. The catheter tip culture showed CRKP in the first 2 days but was negative from the third day to the ninth day, and the urine culture was negative starting on the fifth day of intermittent catheterization. After 11 days of rehabilitation treatment, the catheter was removed, and the patient urinated successfully with a urine volume of 230-330 ml each time. Both the patient’s urine WBC and blood CRP levels returned to normal ranges, as did her body temperature (**Figure 1**). The patient was discharged on the 3rd day after catheter removal, and the routine urinalyses were persistently normal at the 4-month follow-up (**Table 1**).

**Table 1:** Results of the routine urinalyses.

	Hospital stays								Follow-up			
	Day 2	Day 8	Day 22	Day 31	Day 33	Day 38	Day 42	Day 52		15-day	3- month	4-month
White blood count (/μL)	706.3	22.4	285.9	50.7	82.6	71.4	127	2.3		ND	ND	ND
Bacterial count (/μL)	24902	23565	16254	78.4	109	114	125	38.9		ND	ND	ND
Nitrite	–	–	+	–	–	–	–	–		–	–	–

ND, no detected; “+” represents positive, “-” represents negative.

## Discussion

The limited therapy strategies for CRKP infections raise more challenges for clinical situations. In particular, catheter-related CRKP infections often cause not only urinary tract infection but also secondary bloodstream infections, which can endanger the life of patients. The formation of bacterial biofilms leads to difficulty in the thorough removal of pathogenic bacteria in catheter-related infections. Thus, the removal of catheters at an early stage is the most important measure for the treatment of urinary tract infections associated with catheterization. In our study, the patient was diagnosed as urinary tract infection by CRKP according to the persistent fever, elevated CRP and the culture of urine. After the removal of the catheter, the patient could not urinate autonomously, resulting in urinary retention. However, long-term indwelling of catheters could easily form biofilms, causing the inability to remove bacteria and an uncontrollable infection, even we used last resource antibiotic class like tigecycline and polymyxin B. Worse still, we found the isolate with high-level resistance to polymyxin B during the therapy without removal of the catheter. On the other hand, bladder irrigation is no longer recommended as a treatment method for urinary tract infection, while cystostomy may destroy the patient’s quality of life with its many complications and high risk of infection (Yu et al., 2012). Promoting autonomous urination is an ideal solution. Intermittent catheterization is widely used in neurogenic bladder training (Zaouter et al., 2009) and is the gold standard for assisting bladder emptying. Intermittent bladder filling and emptying contribute to recovery of the bladder reflex. Although the patient did not match the diagnosis of neurogenic bladder, we extended the application range of intermittent catheterization. The purpose was to shorten the indwelling time of each catheter, preventing the formation of bacterial biofilm and promoting the control of infection sources, as well as training the bladder function. A catheter with a 12CF diameter was used to minimize the urethral injury. The outcome of this case indicated that intermittent catheterization was effective in removing biofilms and helpful for the control of urinary tract infections.

Acupuncture is an effective method to reduce urinary retention and urinary tract infection and shorten hospital stays (Zaouter et al., 2009; Yu et al., 2012). A previous study demonstrated that acupuncture can significantly improve urinary retention safely and effectively (Chen et al., 2020). After system evaluation, rehabilitation acupuncture doctors performed acupuncture treatment on this patient, including selected acupoints of San Yinjiao, Middle Pole, Guan Yuan, Zu Sanli, and Qi Hai, which can be beneficial to the function of the viscera while using traditional Chinese medicine theory and can contributing to autonomous urination. Pelvic floor muscle function training can improve the muscle strength of the anal sphincter, urethral sphincter and pelvic sacral muscle of patients, and can increase the urethral orifice resistance and the support function of the pelvic floor muscle to promote urination. At the same time, the patient was instructed to lift her chest, pull in her abdomen and lift her buttocks to exercise her lumbosacral muscles and pelvic floor muscles to promote the recovery of the coordination between the bladder detrusor muscle and the urethral sphincter. In the first 2 days after catheter removal, the Crede manual pressure method was used to promote urination; the patient or her family members were taught to put their hands above the pubic bone and forcefully press the lower abdomen to squeeze urine out of the bladder (Stöhrer et al., 2009). After removal of the catheter, the patient could urinate autonomously without the need for resetting the catheter, contributing to the control of infection, and the residual urine volume was between 50-80 ml during the follow-up.

To our knowledge, the successful application of intermittent catheterization combined with rehabilitation in the treatment of catheter-associated CRKP urinary tract infection was rarely reported. As there is an increment in the reports of carbapenem-resistant strains in other groups (Hong et al., 2015; Molina-Mora et al., 2020; Gottesman et al., 2021; Molina-Mora and García, 2021) with limited therapeutic strategies, non-medicine methods like intermittent catheterization deserve further investigation.

## Conclusion

At present, systemic antibiotics are still the most commonly used method for catheter-associated CRKP urinary tract infections. Most studies have focused on oral or parenteral anti-infective therapy, while data on anti-infective therapy are limited. In this case, in addition to a rational application of antibiotics, intermittent catheterization was used to remove catheter bacterial biofilm, which was conducive to infection control. For patients with urinary retention, the recovery of autonomous urination is the premise. Acupuncture treatment and strengthening the pelvic floor muscle through training can promote autonomous urination and ultimately improve the cure rate of CRKP catheter-related urinary tract infections. Functional exercise further promotes the coordination between pelvic floor muscles and the bladder and improves urination function in the long term. Therefore, intermittent catheterization combined with rehabilitation adjuvant therapy provides a new approach for the treatment of CRKP catheter-related urinary tract infections.

## Data availability statement

The original contributions presented in the study are included in the article/supplementary material. Further inquiries can be directed to the corresponding author.

## Ethics statement

The studies involving human participants were reviewed and approved by Sir Run Run Shaw Hospital, Zhejiang University School of Medicine (No. 2022-571-01). The patients/participants provided their written informed consent to participate in this study.

## Author contributions

LS and HW contributed to both in the conception and design of the study. HC and LS organized the data together. HW wrote the first draft of the manuscript. LS and YJ helped perform the analysis with constructive discussions. All authors contributed to manuscript revision, read, and approved the submitted version.

## Funding

This work was supported by the Natural Science Foundation of Zhejiang Province, China (No. LY22H190001) and the China International Medical Foundation for Young Scientists (No. Z-2018-35-2003).

## Conflict of interest

The authors declare that the research was conducted in the absence of any commercial or financial relationships that could be construed as a potential conflict of interest.

## Publisher’s note

All claims expressed in this article are solely those of the authors and do not necessarily represent those of their affiliated organizations, or those of the publisher, the editors and the reviewers. Any product that may be evaluated in this article, or claim that may be made by its manufacturer, is not guaranteed or endorsed by the publisher.

## References

[B1] Chen S. Sun H. Xu H. Zhang Y. Wang H. (2020). Effects of acupuncture on hospitalized patients with urinary retention. Evidence-Based Complement Altern. Med. 2020, 1–7. doi: 10.1155/2020/2520483 PMC6995310 32051686

[B2] Costerton J. W. Stewart P. S. Greenberg E. P. (1999). Bacterial biofilms: A common cause of persistent infections. Sci. (American Assoc. Adv Sci) 284 (5418), 1318–1322. doi: 10.1126/science.284.5418.1318 10334980

[B3] Gottesman T. Fedorowsky R. Yerushalmi R. Lellouche J. Nutman A. (2021). An outbreak of carbapenem-resistant acinetobacter baumannii in a COVID-19 dedicated hospital. Infect. Prev. Pract. 3 (1), 100113. doi: 10.1016/j.infpip.2021.100113 34316574 PMC7794049

[B4] Hong D. J. Bae I. K. Jang I. H. Jeong S. H. Kang H. K. Lee K. (2015). Epidemiology and characteristics of metallo-β-Lactamase-Producing pseudomonas aeruginosa. Infect. Chemother. 47 (2), 81–97. doi: 10.3947/ic.2015.47.2.81 26157586 PMC4495280

[B5] Hu F. Chen S. Xu X. Guo Y Liu Y Zhu D. (2012). Emergence of carbapenem-resistant clinical enterobacteriaceae isolates from a teaching hospital in shanghai, China. J. Med. Microbiol. 61 (1), 132–136. doi: 10.1099/jmm.0.036483-0 21903823

[B6] Miranda E. Boillat C. Kherad O. (2017). Sonde urinaire : respecter les indications pour éviter les infections [Urinary catheter : comply with guidelines to avoid infections]. Rev. Med. Suisse 13 (547), 273–275. 28704006

[B7] Molina-Mora J. A. Campos-Sánchez R. Rodríguez C. Shi L. García F. (2020). High quality 3C *de novo* assembly and annotation of a multidrug resistant ST-111 pseudomonas aeruginosa genome: Benchmark of hybrid and non-hybrid assemblers. Sci. Rep. 10 (1), 1392. doi: 10.1038/s41598-020-58319-6 31996747 PMC6989561

[B8] Molina-Mora J. A. García F. (2021). Molecular determinants of antibiotic resistance in the Costa Rican pseudomonas aeruginosa AG1 by a multi-omics approach: A review of 10 years of study. Phenomics 1 (3), 129–142. doi: 10.1007/s43657-021-00016-z 35233560 PMC8210740

[B9] Podschun R. Ullmann U. (1998). Klebsiella spp. as nosocomial pathogens: epidemiology, taxonomy, typing methods, pathogenicity factors. Clin. Microbiol. Rev. 11, 589–603. doi: 10.1128/CMR.11.4.589 9767057 PMC88898

[B10] Stöhrer M. Blok B. Castro-Diaz D. Chartier-Kastler E. Del Popolo G. Kramer G.. (2009). EAU guidelines on neurogenic lower urinary tract dysfunction. Eur. Urol 56 (1), 81–88. doi: 10.1016/j.eururo.2009.04.028 19403235

[B11] van Duin D. Kaye K. S. Neuner E. A. Bonomo RA. (2013). Carbapenem-resistant enterobacteriaceae: A review of treatment and outcomes. Diagn. Microbiol. Infect. Dis. 75 (2), 115–120. doi: 10.1016/j.diagmicrobio.2012.11.009 23290507 PMC3947910

[B12] WHO (2017). Guidelines for the prevention and control of carbapenem-resistant enterobacteriaceae, acinetobacter baumannii and pseudomonas aeruginosa in health care facilities (Geneva: World Health Organization). 29630191

[B13] Yu K. W. Lin C. L. Hung C. C. Chou E. C. Hsieh Y. L. Li T. M.. (2012). Effects of electroacupuncture on recent stroke inpatients with incomplete bladder emptying: a preliminary study. Clin. Interv Aging 7, 469–474. doi: 10.2147/CIA.S37531 23152677 PMC3496194

[B14] Zaouter C. Kaneva P. Carli F. (2009). Less urinary tract infection by earlier removal of bladder catheter in surgical patients receiving thoracic epidural analgesia. Reg Anesth. Pain Med. 34 (6), 542–548. doi: 10.1097/AAP.0b013e3181ae9fac 19916208

[B15] Zhao F. Zhang J. Fu Y. Ruan Z Xie X. (2015). Dissemination of extensively drug-resistant and KPC-2 producing klebsiella pneumoniae isolated from bloodstream infections. J. infect dev ctries 9 (9), 1016–1021. doi: 10.3855/jidc.6679 26409744

